# Risk factors of sleep-disordered breathing in haemodialysis patients

**DOI:** 10.1371/journal.pone.0220932

**Published:** 2019-08-12

**Authors:** Ginger Chu, Belinda Suthers, Luke Moore, Gemma M. Paech, Michael J. Hensley, Vanessa M. McDonald, Peter Choi

**Affiliations:** 1 Department of Nephrology, Medical & Interventional Services, John Hunter Hospital, Hunter New England Local Health District, New South Wales, Australia; 2 School of Nursing and Midwifery, University of Newcastle, New South Wales, Australia; 3 Research, Innovation and Partnerships, Hunter New England Local Health District, New South Wales, Australia; 4 Priority Research Centre for Healthy Lungs, University of Newcastle, New South Wales, Australia; 5 School of Medicine and Public Health, University of Newcastle, New South Wales, Australia; 6 Department of Respiratory & Sleep Medicine, Medical & Interventional Services, John Hunter Hospital, Hunter New England Local Health District, New South Wales, Australia; IRCCS Istituto Delle Scienze Neurologiche di Bologna, ITALY

## Abstract

**Background:**

Sleep-disordered breathing (SDB) is common in patients with kidney disease; but often underdiagnosed as it is infrequently assessed in clinical practice. The objective of this study was to assess the risk factors of SDB in haemodialysis patients, and to identify useful assessment tools to detect SDB in this population.

**Methods:**

We used nocturnal oximetry, Epworth Sleepiness Scale (ESS) and STOPBANG questionnaire to screen for SDB in haemodialysis patients. Presence of SDB was defined by Oxygen desaturation index (ODI≥5/h), and further confirmed by apnoea-hypopnea index (AHI) from an in-laboratory polysomnography. Blood samples were collected prior to commencing a haemodialysis treatment.

**Results:**

SDB was detected in 70% of participants (N = 107, mean age 67 years). STOPBANG revealed that 89% of participants were at risk of SDB; however, only 17% reported daytime sleepiness on the ESS. Of the participants who underwent polysomnography (n = 36), obstructive sleep apnoea was identified in 86%, and median AHI was 34.5/h. Oximetry and AHI results were positively correlated (r = 0.62, P = 0.0001), as were oximetry and STOPBANG (r = 0.48; P<0.0001), but not ESS (r = 0.19; P = 0.08). Multivariate analysis showed that neck circumference (OR: 1.20; 95% CI: 1.07–1.34; P = 0.02) and haemoglobin (OR: 0.93; 95% CI: 0.88–0.97; P = 0.003) were independently associated with the presence of SDB.

**Conclusion:**

Dialysis patients with a large neck circumference and anaemia are at risk of SDB; using nocturnal oximetry is practical and reliable to screen for SDB and should be considered in routine management of dialysis patients, particularly for those who demonstrate risk factors.

## Introduction

Sleep-disordered breathing (SDB) is a common cause of sleep disturbance in patients receiving haemodialysis. Previous studies have indicated that more than 50% of dialysis patients experience SDB [1]. This is concerning as the prevalence in this population is five times that of healthy male Australians aged over 45 years [2]. Despite this, currently there is no clinical guideline/recommendation on either the screening or management of SDB in the dialysis population, and perhaps unsurprisingly assessment of SDB in patients with kidney disease is seldom performed in clinical practice. Patients with chronic kidney disease (CKD) already suffer from a high symptom burden [3] and impaired quality of life (QoL); accordingly managing SDB may reduce the impact of comorbidities and improve QoL in this population.

SDB contributes to significant morbidity and mortality in patients with CKD [4, 5]. The Sleep Heart Health Study demonstrated that SDB is independently associated with cardiovascular disease [6], which is one of the leading causes of death in patients receiving dialysis [7]. As such, screening for SDB appears an important but under performed assessment.

Screening SDB can be conducted using validated subjective measures such as the STOPBANG questionnaire [8] and Epworth sleepiness scale (ESS) [9]; however clinical diagnosis still requires polysomnography (PSG) [10]. An in-laboratory attended PSG is the gold standard to diagnose SDB. The test is traditionally performed in a sleep centre and hence can be expensive and onerous for patients, particularly for those with chronic and complex diseases with already high healthcare requirement. Furthermore, access is often difficult; the average waiting time for a non-urgent in-laboratory PSG in Australia is up to 68 weeks [11]. Therefore, an alternative objective assessment of SDB using nocturnal oximetry has increasingly been proposed for clinical practice [11].

To date, there are few studies using nocturnal oximetry to detect SDB in the haemodialysis population. There is also insufficient evidence evaluating approaches that can effectively screen for SDB in this population; one that is known to have high prevalence of SDB. In this context, we aimed to evaluate the clinical utility of screening tools, and to identify risk-factors associated with SDB in a population of patients with chronic kidney disease receiving dialysis.

## Materials and methods

### Study design and participants

For this prospective cross-sectional study, patients receiving haemodialysis at four metropolitan dialysis units in the Hunter Region of New South Wales, Australia between February and December 2017 were screened for suitability. Eligible participants were ≥18 years of age, had satisfactory English language skills and had received stable haemodialysis for more than three months. The exclusion criteria were acute or home dialysis, inpatient hospitalisation, and prior treatment for SDB.

This trial was approved by Hunter New England Local Health District Human Research Ethics Committee (16/09/21/4.02) and the University of Newcastle Human Research Ethics Committee (Dev-004267). The study was conducted according to the principles of Good Clinical Practice and all participants provided written informed consent.

#### Clinical data

The baseline data included patient demographics, dialysis characteristics, and sleep-related questionnaires (Table 1). Objective sleep data included nocturnal oximetry and in-lab PSG (for patients with an abnormal oximetry); both tests were performed on the night after a dialysis session to minimize the influence of fluid overload on respiratory function. All blood samples were collected pre-dialysis and analysed at the local pathology laboratory using standard methods (Pathology North, John Hunter Hospital, Newcastle, Australia). Dialysis adequacy was measured by machine-calculated single pool Kt/V.

**Table 1:** Characteristics of participants.

Demographic	Screening	Polysomnography
Total participants (n)	107	36
Age (years)	67±15	65±15
Sex		
Men, n (%)	62 (58%)	26 (72%)
Women, n (%)	45 (42%)	10 (28%)
Dialysis vintage (m)	57±52	44±30
BMI (kg/m^2^)	29.1±7.2	32.2±6.5
Average spKt/V	1.5 ± 0.3	1.3±0.3
Average inter-dialytic weight gain (L)	1.9 ± 1.2	2.4±1.2
Smoking, n (%)	6 (6%)	2 (6%)
Hypertension, n (%)	71 (66%)	22 (61%)
Diabetes, n (%)	45 (42%)	18 (50%)
Type 1	9 (20%)	4 (22%)
Type 2	36 (80%)	14 (78%)
Heart failure, n (%)	31 (29%)	14 (39%)
Stroke, n (%)	17 (16%)	7 (19%)
Malignancy, n (%)	17 (16%)	6 (17%)
Mental health disorder, n (%)	32 (30%)	11 (31%)
Working	11 (10%)	4 (11%)
Work hours (per week)^1^	0 (0,0)	0 (0, 0)
Shift worker	7 (7%)	2 (6%)
**Sleep-Related Data**		
ESS, score	6.3±4.4	7.3±3.7
STOPBANG, score	4.5±1.5	5.2±1.4
Snoring, n (%)	64 (60%)	28 (78%)
Neck circumferences (cm)	43.2±6.3	46.1±5.4
ODI, score^1^	9.7 (4, 27.5)	26.5 (11.6, 37)
Spo2	92.3±3.0	95.1±2.8
Lowest Spo2	75.3±11.1	73.4±9.9
% time Spo2<90^1^	7.8 (0.8, 27.1)	13.7 (5.6, 44)
AHI, score^1^		34.5 (21.8, 61.3)
TST^1^ (minutes)		388 (276, 438)
WASO^1^ (minutes)		93 (45, 140)
Sleep Stages (% TST)^1^		
Stage 1		11.4 (4.6, 24.1)
Stage 2		59.3 (42.2, 78.1)
Stage 3		11.3 (5.4, 26.0)
REM		9.5 (5.2, 13.9)
SE (%)		71±18
Arousals (/h)		28.2±19.8
**Biochemical Results**		
Haemoglobin (g/L)	111.2 ± 13.5	108.0±15.5
Sodium (mmol/L)	137.8 ± 3.2	137±2.6
Potassium (mmol/L)	5.0 ± 0.7	5.0±0.8
Chloride (mmol/L)	101.6 ± 4.5	100.5±3.1
bicarbonate (mmol/L)	22.7 ± 3.6	23.1±2.9
Urea(mmol/L)	18.4 ± 5.5	19.3±7.2
Calcium (mmol/L)	2.3 ± 0.3	2.3±0.2
Phosphorus (mmol/L)	1.7 ± 0.6	1.8±0.7
Albumin (g/L)	32.0 ± 4.3	32.6±4.2
Corrected Calcium (mmol/L)	2.4 ± 0.2	2.5±0.2

Abbreviations: BMI, body mass index; ESS, Epworth sleepiness scale; ODI, overnight desaturation index; AHI, apnoea-hypopnea index; SpO2, peripheral capillary oxygen saturation; TST, total sleep time; WASO, wake after sleep onset; REM, rapid eye-movement; SE, sleep efficiency.

Continuous variables are expressed as mean (±standard deviation) or median (25^th^-75^th^ percentile)

^1^; categorical variables are presented as number (percentage).

#### Nocturnal oximetry

Overnight oximetry was performed by a transmission technology oximeter (Nonin 3150 WristOx_2_ Pulse Oximeter; Nonin Medical Inc., MN, USA), a type four device that is widely used as a screening tool for SDB [12]. Participants were asked to apply the oximetry sensor to the index finger overnight during sleep. Sleep and wake time were completed by the participants, and a total sleep time less than four hours was considered an incomplete test. The records obtained were retrieved by the same sleep technician (LM) who was blinded to other assessment methods, and analysed using the PROFOX software (PROFOX Oximetry Version Masimo; PROFOX Associates Inc., PA, USA). The oxygen desaturation index (ODI) is calculated as the number of drops in SpO2 ≥ 3% below the baseline oxygen level with a sampling rate of 2 seconds, and ODI ≥5/hour was considered abnormal.

#### Polysomnography

An in-laboratory PSG, type one device (Compumedics Grael 4K PSG plus; Compumedics Limited, Abbotsford, Australia), was used to measure sleep stages and respiratory events. It combines signals from electroencephalography, electromyography, electro-oculography, electrocardiography, nasal airflow, and respiratory effort (dual thoracoabdominal respiratory inductance plethysmography belts), and monitors respiratory events against sleep stages and body positions [13]. The records obtained were analysed using Compumedics Profusion 4 software (Compumedics Limited, Abbotsford, Australia), and respiratory events were manually scored by the same sleep technician (GP), who was blinded to other assessments, using American Academy of Sleep Medicine (AASM) 2017 scoring rules [14]. The final results were reported by the sleep physician of the sleep centre. We followed the Australasian Sleep Association definition of an apnoea-hypopnea index (AHI) of 5–14.9 events/hour as mild SDB; 15–29.9 being moderate; and ≥30 being severe [10].

#### Patient-reported questionnaires

Questionnaires were delivered to all eligible participants and collected by the same author (GC). Participants completed the Epworth Sleepiness Scale [9] and the STOPBANG questionnaire [8] prior to attending nocturnal oximetry. Patients were encouraged to reflect on their overall sleepiness in the previous month rather than on the day of completing the survey.

#### Outcomes and sample size

The co-primary outcomes were risk factors of SDB and appropriateness of screening tools. Samples were drawn from the local dialysis service that has approximately 200 haemodialysis patients, and a sample of 100 would allow the study to detect a clinically relevant Area Under the Curve (AUC) of 0.85 and 95% confidence interval with a margin of error of at least 10%.

### Analysis

Statistical analyses were performed using Stata version 15 (Stata Corporation, College Station, Texas, USA). Continuous variables are reported as mean and standard deviation (±SD) or median and interquartile range (IQR), depending on normality. Pearson’s correlation coefficient was used to assess the correlation between continuous variables. For assessing risk factors, logistic regression was used to determine the relationship between binary outcomes (presence vs absence of SDB) and dependent variables. Variables that have statistical and clinical significance were entered into a multivariate analysis. All significance tests were two-sided and a statistical value of p <0.05 was considered statistically significant.

## Results

### Participant characteristics

Eligible participants (N = 107) had undertaken questionnaires and 93 completed nocturnal oximetry assessment. Only nine patients were excluded because of prior identification of SDB. Participants’ baseline characteristics are summarized in Table 1. In brief, the 107 participants had a mean age of 67 years, 58% were male, and their mean body mass index (BMI) was 29.1kg/m^2^. Hypertension was prevalent in 66% and diabetes mellitus in 42% (80% type II) of the participants. Coexisting periodic limb movements (PLM) was evident in 28% of patients with sleep apnoea who underwent a PSG, and the mean PLM index was 66/h.

### Characteristics of sleep-disordered breathing

The results of overnight oximetry demonstrated that 70% of participants had SDB, and 89% were identified as at risk by STOPBANG questionnaire. The mean ±SD STOPBANG score was 4.5±1.5, and 60% of participants reported regular snoring. Despite the high rates of abnormal overnight oximetry and STOPBANG scores, only 17% of patients reported daytime sleepiness on ESS, with mean ESS score of 6.3±4.4.

For patients with an abnormal ODI (n = 65, 70%), 36 underwent a formal PSG, and the median (IQR) AHI score was 34.5/h (21.8, 61.3) confirming severe sleep apnoea. The proportion of participants classified with mild, moderate and severe sleep apnoea were 5 (14%), 12 (33%) and 19 (53%), respectively. The majority had obstructive sleep apnoea (84%), with only a small proportion showing a mixture of obstructive and central sleep apnoea (16%). We did not perform statistical analysis of association between abnormal AHI scores and sleep questionnaire or patient characteristics because only a small number of participants underwent PSG.

### Risk factors and performance of screening tools

Univariate regression showed that BMI, neck circumference, average Kt/V, diabetes and haemoglobin were associated with SDB (Table 2). In multivariate analysis, neck circumference and haemoglobin remained independently associated with SDB (Table 3).

**Table 2:** Univariate analysis of predictive factors of all screened participants for the presence of sleep-disordered breathing (defined by ODI≥5).

Variable			
	OR	95% CI	p Value
**Demographic**			
Male (vs female)	1.70	0.69–4.20	0.12
Age (years)	1.00	0.97–1.03	0.98
BMI (kg/m^2^)	1.13	1.04–1.23	0.03*
Neck circumferences (cm)	1.15	1.06–1.25	0.001*
Smoking (vs non-smoking)	0.42	0.08–2.24	0.29
Sedative (vs non-sedative medication)	2.75	0.31–23.94	0.36
**Dialysis**			
Vintage (months)	1.00	0.99–1.00	0.30
Afternoon (vs morning shift)	2.02	0.71–5.72	0.19
HDF (vs HD)	0.86	0.35–2.08	0.73
Average kt/v	0.17	0.04–0.66	0.01*
Average interdialytic weight gain (L)	1.11	0.76–1.62	0.60
**Comorbidity**			
Diabetes	3.09	1.16–8.28	0.02*
Hypertension	0.68	0.26–1.79	0.44
Heart Failure	2.30	0.82–6.43	0.12
History of Stroke	0.95	0.30–3.06	0.91
Cancer	0.51	0.16–1.65	0.27
Mental health disorder	1.01	0.39–2.61	0.99
**Hematologic and Biochemical parameters**			
Haemoglobin (g/L)	0.95	0.91–0.99	0.01*
Sodium (mmol/L)	0.96	0.84–1.09	0.52
Calcium (mmol/L)	0.54	0.10–2.94	0.47
Corrected calcium (mmol/L)	0.68	0.10–4.91	0.74
Phosphorus (mmol/L)	0.88	0.41–1.86	0.64
Albumin (g/L)	0.94	0.84–1.05	0.20
Urea (mmol/L)	1.03	0.95–1.12	0.50
Bicarbonate (mmol/L)	1.06	0.92–1.23	0.42
Potassium (mmol/L)	0.96	0.51–1.79	0.88

N = 107. Each comorbidity versus others (e.g. diabetes vs non-diabetes). Hematologic and biochemical parameter is per one unit increase. Abbreviations: OR, odds ratio; CI, confidence interval.

*P<0.05.

**Table 3:** Multivariate analysis of predictive factors for presence of sleep-disordered breathing (defined by ODI≥5).

Variable	Unit of increase	OR	95% CI	p Value
Neck Circumferences	1 cm	1.20	1.07–1.34	0.02*
Haemoglobin	1 g/L	0.93	0.88–0.97	0.003*
Diabetes	Diabetes vs no	2.84	0.80–10.02	0.11
Heart Failure	Heart Failure vs no	1.45	0.44–4.93	0.74
Hypertension	Hypertension vs no	0.84	0.27–2.64	0.42
IDWG	1 kg	0.79	0.47–1.31	0.27
Kt/v	1 unit	0.12	0.01–1.17	0.07
Age	1 year	0.99	0.95–1.03	0.21
Sex	Male	0.96	0.26–3.46	0.53

N = 107. AUC = 0.83, CI: (0.74–0.91), P = 0.04. Abbreviations: OR, odds ratio; CI, confidence interval; IDWG, interdialytic weight gain; AUC, area under the curve.

*P<0.05.

ODI was well correlated with STOPBANG (r = 0.48; P<0.0001), but not ESS (r = 0.19; P = 0.08). In the subset of patients who performed oximetry and PSG, ODI correlated strongly with AHI (r = 0.62, P = 0.0001).

## Discussion

We used nocturnal oximetry and in-lab PSG to determine the presence and the severity of SDB in a representative sample of patients receiving maintenance haemodialysis. We report that the presence of SDB as measured by overnight oximetry is high at 70%, having excluded patients with a prior diagnosis of SDB. In those patients who underwent PSG, 81% had moderate to severe sleep apnoea. Interestingly, while fatigue and excessive daytime sleepiness (EDS) are commonly reported by haemodialysis patients, the prevalence of EDS measured using a validated questionnaire was low; less than expected from the extent and severity of SDB. We found risk factors that were associated with SDB included large neck circumference and anaemia.

The STOPBANG questionnaire detected higher prevalence (89%) of SDB compared with nocturnal oximetry. Nicholl *et al*. examined a mixed population of patients with kidney disease (CKD: n = 109 and End Stage Kidney Disease: n = 63) and demonstrated that a STOPBANG score of ≥3 provided 93% sensitivity in identifying the risk of SDB in this population. However, due to the large proportion of kidney disease patients being elderly and having hypertension, the specificity of this screening tool was only 29% [15]. To overcome the low specificity for detection of SDB using STOPBANG questionnaire, we suggest that these previously published data and our present study support the use of objective measures to accurately identify SDB in kidney disease population.

Fatigue and sleepiness are commonly observed in patients with kidney disease when symptom-screening questionnaires are applied [16]. We measured excessive daytime sleepiness using the ESS, expecting to observe a high prevalence of EDS. However, we found that the rate of self-reported daytime sleepiness was relatively low and was not correlated to the presence of SDB. This is important as SDB is infrequently assessed in clinical practice in patients with kidney disease, and the low report of EDS in this population will further limit assessment due to an absence of ‘red flags’. The inconsistency of ESS and SDB in patients with kidney disease was reported by Roumelioti *et al* who noted that the level of EDS was inconsistently correlated with the presence of SDB (r = 0.18, p = 0.13 for central sleep apnoea and r = 0.27, p = 0.02 for obstructive sleep apnoea) [17]. Why then should the ESS fail to correlate with measures of SDB, when SDB is known to cause daytime sleepiness in the general population? A possibility is that ESS is subjective, and patients’ estimation of their own degree of sleepiness can be influenced by factors such as being accustomed to chronic fatigue, disease denial, or employment pressure, particularly for commercial drivers [18, 19]. Another possibility is that patients with ESKD interpret the ESS questions in a manner that mitigates against sensitivity. The majority of participants in this study were elderly and unemployed or retired. Many report having regular daytime naps and/or sleeping through their dialysis treatments. EDS may not be noticed by patients in this context. Overall, our study highlighted the deficiencies of using the self-reported ESS as a surrogate for SDB in the haemodialysis population.

We performed nocturnal oximetry in relatively large number of patients with ESKD and identified a number of risk factors that were independently associated with SDB, including the presence of large neck circumference, and anaemia. We found that large neck circumference was a better predictor of SDB than BMI in haemodialysis patients. Beecroft *et al*. demonstrated that haemodialysis patients had reduced upper airway volume compared with patients without kidney disease of similar BMI and suggested that the decreased airway size was driven by fluid retention rather than fat deposition [20]. The impact of fluid retention on SDB was reported in a cross-sectional study of 43 haemodialysis patients, with those with sleep apnoea having higher total body extracellular fluid volume (ECFV) (17.7±2.9L vs.15.1±2.6L, p = 0.006), and higher evening ECFV in the leg (p = 0.010), throat (p = 0.028) and neck (p = 0.016) compared with patients without sleep apnoea [21]. We suggest that these data should lead to prospective studies to determine whether the fluid accumulation affects pharyngeal size and if management strategies on fluid removal may aid haemodialysis patients with large neck circumferences and SDB. In the multivariate analysis, we also found that anaemia was strongly associated with the presence of SDB. Haemoglobin plays a substantial role in respiratory function by carrying oxygen throughout the body. The pathophysiology link between anaemia and SDB has not been published previously and is not fully understood. Given that anaemia is a common symptom of patients with kidney disease, we speculate that our observed association of SDB and anaemia is a reflection of complex bidirectional relationship between SDB and CKD [22], and that in clinical practice when a patient presents with one condition they should be considered for the other.

The novel clinical implication of this study is that the high rates of unrecognized SDB suffered by haemodialysis patients can be identified by non-invasive nocturnal oximetry performed in patients’ homes. This clinical strategy provides more accurate clinical data than patient-reported questionnaires, but is less cumbersome and expensive than formal PSG. During the conduct of this study, patients anecdotally reported a high level of acceptability for home oximetry devices, which were self-applied to patients’ wrists prior to sleep, and returned by the patient at their next scheduled dialysis visit. Nocturnal oximetry correlated well with formal PSG and given the potential overestimation of STOPBANG in haemodialysis, nocturnal oximetry is a useful tool to identify SDB in those patients who were considered as a high risk from STOPBANG questionnaire.

Our study has several strengths. Firstly, data were collected prospectively, according to a pre-specified, hypothesis-driven protocol applied to a discrete haemodialysis population receiving similar care. Secondly, we achieved a high rate of recruitment, with 107 of 117 (91%) of eligible patients providing data. We achieved universal measurement of ODI, STOPBANG and ESS in comparison to previous studies which have either predominately relied upon self-reported questionnaires [23–25], had a small sample size [1, 26] or included non-dialysis CKD populations [27]. The largest prior studies by Tada *et al*. and Ogna *et al*. did not examine the use of nocturnal oximetry in screening for SDB in haemodialysis patients [28, 29]. To the best of our knowledge, this is the largest cohort study analysing nocturnal oximetry and risk factor of SDB in an Australian haemodialysis population.

There are also some limitations that need to be considered. Firstly, although most eligible participants were recruited, 54 (32%) of local dialysis patients were ineligible at the initial recruitment due to exclusion criteria, and this exclusion had the potential to introduce a selection bias. However, the majority of patients were excluded due to poor health, poor cognitive status or prior treatments for SDB, we suggest that the excluded patients have not invalidated our finding of a high presence of SDB, since they might be expected to show a high rate of SDB. In addition, nocturnal oximetry and PSG were conducted post dialysis when the fluid accumulation was at its minimal, which could also mitigate the presence of SDB. Secondly, we only performed an in-laboratory PSG on approximately 55% of participants with abnormal nocturnal oximetry (n = 36/65), while another 45% either declined to attend the test or were unable to due to social/physical circumstances. Since PSG was only performed in patients who had abnormal oximetry, this study is unable to validate the negative predictive power of oximetry in patients with normal ODI. However, given that in-laboratory PSG is expensive, burdensome to patients, referring patients who are not at risk of SDB to an in-laboratory PSG assessment may not be the best clinical practice [30]. The cross-sectional design of our study does not allow us to infer causality based on the association between SDB and risk factors. Either a randomised controlled trial of an intervention to correct a risk factor or a longitudinal study prior to development of SDB are required to examine a causal relationship between risk factors and SDB.

In conclusion, our data indicates that the presence and severity of SDB is high in haemodialysis patients. Patients with SDB may not present with excessive daytime sleepiness. Assessing SDB using nocturnal oximetry is adequate in identifying haemodialysis patients at high risk of SDB and should be considered in the routine assessment and management of this population.

## Supporting information

S1 Fig Recruitment diagram. Flowchart showing the selection process for the study participants.
(TIF)

S2 Fig Correlation between nocturnal oximetry and other sleep measures. Pearson Correlation between ODI and AHI, STOPBANG and ESS.
(TIF)
