# Supplementary figures and images for: Risk factors of sleep-disordered breathing in haemodialysis patients
Source: PLoS One. 2019 Aug 12;14(8):e0220932. doi: 10.1371/journal.pone.0220932 (PMC6690564; doi:10.1371/journal.pone.0220932)

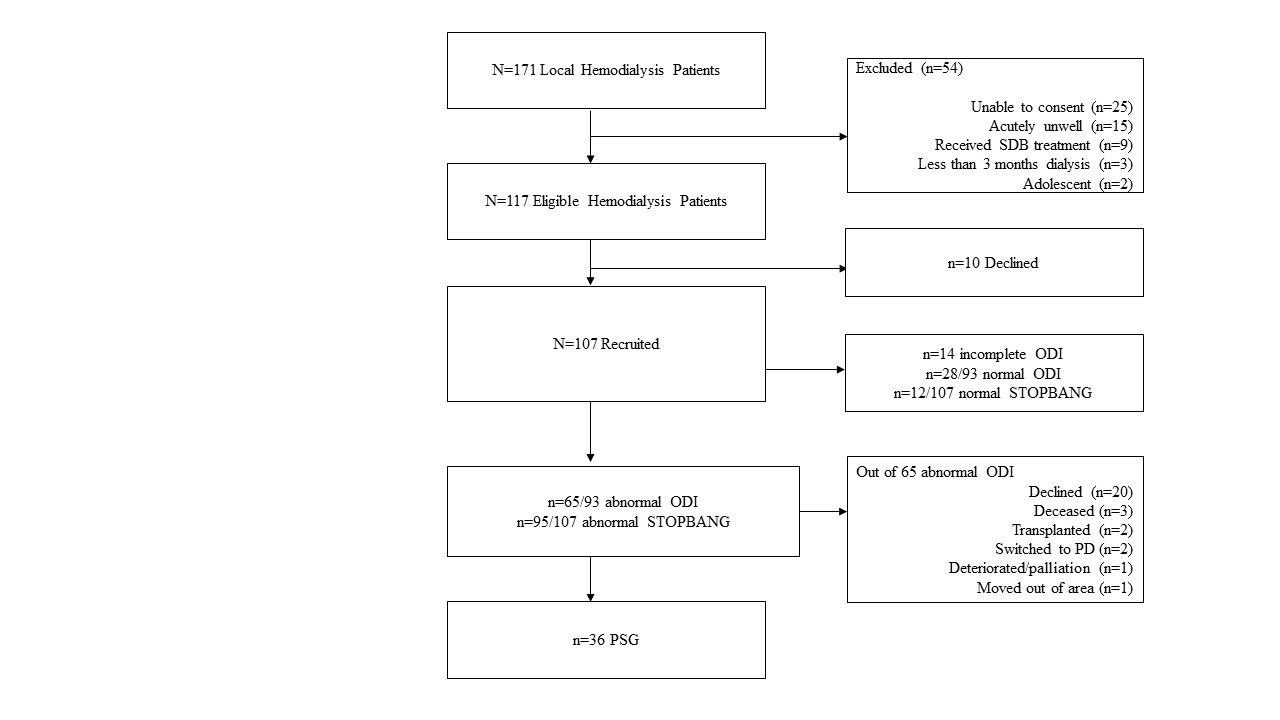

Supplement: S1 Fig — Flowchart showing the selection process for the study participants. (TIF) [file pone.0220932.s001.tif]

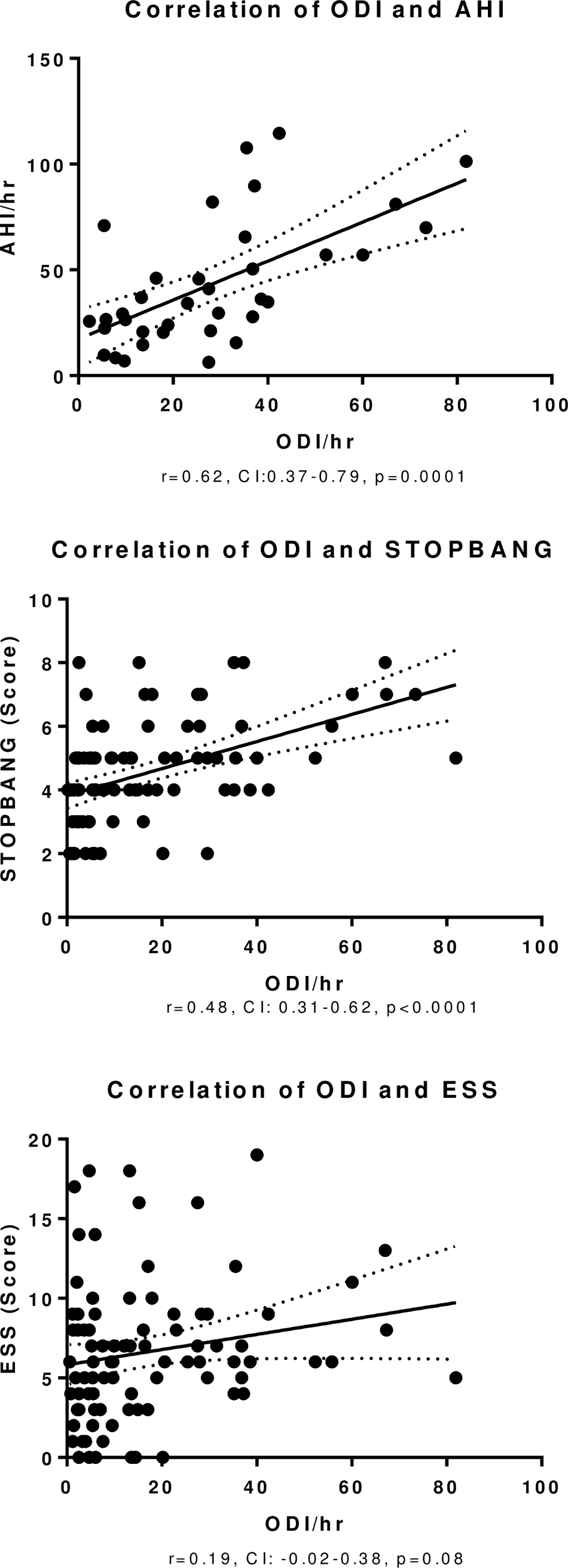

Supplement: S2 Fig — Pearson Correlation between ODI and AHI, STOPBANG and ESS. (TIF) [file pone.0220932.s002.tif]
